# Addition of clidinium-C to the 14-day proton-pump inhibitor-based triple therapy for Helicobacter pylori eradication

**Published:** 2016

**Authors:** Mohammadreza Seyyedmajidi, Saba Homapoor, Elahe Zanganeh, Mohammad Dadjou, Shahab Eskandari Nejad, Mohammad Hadi Tajik Galayeri, Jamshid Vafaeimanesh

**Affiliations:** 1 Golestan Research Center of Gastroenterology and Hepatology-GRCGH, Gorgan, Iran.; 2 Clinical Research Development Center, Qom University of Medical Sciences, Qom, Iran.

**Keywords:** Clidinium-C, Helicobacter pylori eradication, Peptic ulcer, Side effects.

## Abstract

**Background:**

Triple therapy with a proton pump inhibitor and two antibiotics in Helicobacter pylori (HP) eradication is widely accepted, but this combination fails in a considerable number of cases. The aim of this study was to assess the effect of clidinium-C addition on HP eradication and to investigate the efficacy and safety of clidinium-C in prevention of drugs' side effects.

**Methods:**

A total of 200 histopathologically confirmed HP positive peptic ulcer enrolled in this study which were randomly assigned to two treatment groups: OAC (20 mg omeprazole bid, 1000 mg amoxicillin bid and 500 mg clarithromycin bid) and OAC + clidinium-C. The effect of treatment and adverse effects were compared 6 weeks after completion of treatment. A13C-urea breath test was performed to confirm HP eradication.

**Results:**

A total of 184 patients (90 in group A and 94 in group B) completed the treatment protocols. HP eradication was achieved in 71.1% in OAC versus 72.3% in OCA+clidinium-C, (P=0.73). The frequencies of abdominal pain and stool abnormality, among the side effects recorded during the therapy period, were significantly lower in group B (OCA+clidinium-C) (P=0.01 and P=0.001, respectively).

**Conclusion:**

Addition of clidinium-C to OCA triple therapy decreases abdominal pain and frequency of stool abnormalities without affecting HP eradication rate. Based on these findings addition of clidinium-C may increase patient's compliance.

Helicobacter Pylori (HP) is a Gram-negative, spiral-shaped, motile micro-organism that infects approximately half of the world population and the prevalence of asymptomatic infected patients appears to be age-related (1). The human is its reservoir and the transmission of this microorganism involves oral‑oral and fecal‑oral routes. The infection takes place usually in childhood, within one’s own family (between parents and children or between siblings) (2). Gastric infection by HP is actually considered to be the most relevant cause of chronic gastritis and peptic ulcer disease (PUD). It is also associated with an increased risk of mucosa associated lymphoid tissue (MALT) lymphoma and gastric cancer (3). Selection of the best drug regimens for effective eradication of HP infection is challenging. Some recent studies have suggested that the effectiveness, compliance and side-effects of quadruple regimen containing a gastric acid inhibitor, a bismuth compound and two antibiotics might be comparable with proton pump inhibitors (PPI) -based triple therapy when administered as first-line treatment for HP infection (4-6). However, in other studies showed superiority of quadruple therapy (7).

Antibiotic resistance due to frequent and uncontrolled administration and high prevalence of antibiotic side effects are the most common causes of treatment failure. To increase eradication rate, as defined in the Maastricht IV report (8), several clinical trials have been initiated involving extended treatment duration, use of new antibiotics or the addition of probiotics or other drugs regimens (9). In the Maastricht Consensus Report, the 14-day treatment course was superior to the 7-day treatment. Because of the higher antibiotic resistance rates, in developing countries such as Iran, the 14-day treatment regimens is preferred. It has been shown that the large doses of antibiotics used in the triple therapy change the normal bowel flora. This may result in further gastrointestinal adverse events (8, 9). Clidinium bromide in combination with chlordiazepoxide (clidinium –C) is an anticholinergic drug which may help symptoms of cramping and abdominal stomach pain by decreasing gastric acid secretion and slowing the intestinal movement (10). Chlordiazepoxide component of this drug exerts anxiolytic, sedative, hypnotic, anticonvulsant and skeletal muscle relaxant effects. The drug may inhibit monosynaptic and polysynaptic reflexes by acting as an inhibitory neuronal transmitter or by blocking excitatory synaptic transmission. This drug may also directly depress motor nerve and muscle function. 

The aim of this study was to assess the effect of clidinium-C on HP eradication with a triple therapy including omeprazole, clarithromycin and amoxicillin (OCA) in patients with peptic ulcer disease (PUD). The secondary aim of the study was to investigate the efficacy and safety of clidinium-C in the prevention of side-effects related to HP eradication.

## Methods

**Patients: **This prospective double-blinded randomized clinical trial study was conducted on 200 consecutive HP infected patients with PUD between March 2011 to November 2012. Subjects were excluded if they were taking non-steroid anti-inflammatory drugs (NSAIDs), PPI, bismuth preparations or antibiotics during the previous eight weeks, pregnant women, age under 18, patients with renal and hepatic impairment or with previous gastric surgery. Gastroscopy was done using a videoscope (Olympus GIF-XQ260, Japan) and two specimens were obtained from the antrum. HP infection was diagnosed by histopathological examination. This research was approved by the Ethics Committee in Golestan University of Medical Sciences. Informed consent was obtained from all patients. IRCT number was 2013041112977N1.

Patients were randomly assigned to one of the two treatment protocols; Group A (control group, n=100): the patients were given a 14-day standard OCA triple therapy for HP infection eradication consisted of 20 mg omeprazole bid, 1000 mg amoxicillin bid and 500 mg clarithromycin bid; Group B (case group, n=100): in this group the patients were given a 14-day clidinium-C bid plus OCA triple therapy. Patients were asked to return at the end of the treatment period. Compliance to treatment was defined as consumption of greater than 80% of the prescribed drugs. Subjects report any side effects of drugs and were given a possible side effect list, such as dyspepsia, nausea, abdominal pain or cramp, stool abnormality, dizziness, headache and bad taste. Medications were discontinued if any intolerable adverse events occurred. A13C-urea breath test was performed for eradication assessment 6 weeks after completion of the treatment. 

Statistical analysis was performed with chi-square test as well as Fisher’s exact test, and one-way analysis of variance (ANOVA) test. P-values of 0.05 or less were considered statistically significant. All the data were analyzed using SPSS Version 16 (SPSS Inc., Chicago, IL, USA) and the values were expressed as mean ± standard deviation (SD) for continuous variables and percentages for categorical variables. 

## Results

One hundred eighty-four of 200 patients (42.9% males, between 19-72 years with mean age of 42.3±11.3 years) completed the study and underwent 13C-urea breath testing: 90 in group A and 94 in group B. There were no statistically significant differences between the two groups regarding age, gender and body mass index (table 1). HP eradication was achieved in 64 of the 90 (71.1%) patients in group A (OCA without clidinium-C) and in 68 of the 94 (72.3%) patients in group B (OCA with clidinium-C). The difference was not statistically significant (P=0.73) (table 2). 

The side effects are shown in table 3. The frequencies of abdominal pain or cramp and stool abnormality, among the side effects recorded during the therapy period, were significantly lower in group B (OCA with clidinium-C) than in group A (P=0.01 and P=0.001, respectively). The differences between the remaining side effects in both groups demonstrated no statistical significance.

**Table 1:** Baseline characteristics of the study subjects in two groups

**Character**	**Groups**		**Total** **(n=184)**	**Pvalue**
	**A** **(Only OCA)** **n=90**	**B** **(CC+OCA)** **n=94**		
Age (years)	42.7±11.9	41.9±10.7	42.3±11.3	0.62
Gender (M/F)	38.52	41.53	79.105	0.81
BMI (Kg/m^2^)	23.6±4.7	24.2±4.5	23.9±4.6	0.43

OCA= omeprazole, amoxicillin and clarithromycin CC=clidinium-C

**Table 2:** Helicobacter pylori eradication rates of study subjects in two groups

**Helicobacter pylori** ** eradication regimens**	**HP eradication rate**	**P-value**
A (Only OCA) (n=90)	64 (71.1%)	0.73
B (CC + OCA) (n=94)	68 (72.3%)		

OCA = Omeprazole, Amoxicillin and Clarithromycin CC = Clidinium-C

**Table 3:** The prevalence of drugs side effects in two groups

** Group** **Characteristics**	**A** **(Only OCA)** **n=90**	**B** **(CC+OCA)** **n=94**	**P-value**
Dyspepsia	13 (14.4)	7 (7.4)	0.21
Nausea	13 (14.4)	6 (6.3)	0.12
Abdominal pain or cramp	9 (10)	1 (1.1)	0.01
Stool abnormality	14 (15.5)	3 (3.1)	0.001
Dizziness	4 (4.4)	5 (5.3)	0.83
Headache	6 (6.6)	5 (5.3)	0.76
Bad taste	18 (20)	19 (20.2)	0.91

OCA = omeprazole, amoxicillin and clarithromycin

CC = clidinium-C

Stool abnormality=changing in form and time of defecation

## Discussion

According to the Maastricht IV Consensus Report about patients with HP infection, in areas of low clarithromycin resistance, clarithromycin-containing treatments are recommended for first-line empirical treatment and bismuth-containing quadruple treatment is also an alternative. In areas of high clarithromycin resistance, bismuth-containing quadruple treatments are recommended for first-line empirical treatment. If this regimen is not available, sequential treatment or a non-bismuth quadruple treatment is recommended. Extending the duration of PPI-clarithromycin-containing triple treatment from 7 to 10-14 days improves the eradication success and may be considered (8).

Antibiotic related side effects during HP eradication are common and usually affect the gastrointestinal system. Poor patient compliance due to the side effects and discontinuation of the therapy impair the efficiency of the therapy and increase the possibility of antibiotic resistance (11, 12). On the other hand, the effect of treatment with clarithromycin containing regimen is decreasing due to a combination of antibiotic resistance and a poor compliance with therapy, which is primarily due to the side effects of the antibiotics (13). In countries in which frequent and uncontrolled antibiotic utilization is common, as in Iran, the HP eradication rates are decreasing. In this study, we selected the use of clidinium-C, because this is an anticholinergic drug which may help symptoms of cramping and abdominal pain. However, to our knowledge there has been no study conducted on the efficacy of clidinium-C on HP eradication

In the present study, the success rate of HP eradication with OCA first-line triple therapy was 71.1% and 72.3% with the addition of clidinium-C (p=0.73). In published studies from western countries, the success rate of HP eradication therapy was reported as approximately 70%, which is near the ideal values (14). Thus, the addition of clidinium-C to conventional regimen had no effect on eradication rate. 

Another goal of our study was to investigate the efficacy of clidinium-C for the prevention of the side effects related to the therapy. It has been shown that the large doses of antibiotics used in the triple therapy change the normal bowel flora. This may explain higher rate of gastrointestinal adverse events in 14-day HP eradication regimen (15). In our study, we did not observe the addition of clidinium-C to OCA regimen to have any effect on the dyspepsia, nausea, dizziness, headache and bad taste in the month. However, abdominal pain and stool abnormality were significantly lower in the clidinium-C arm than in control group. This effect is important for those countries in which HP eradication regimens are applied for longer periods (10-14 day).

In Conclusion, the study suggests that the addition of clidinium-C to OCA triple therapy significantly decreases the frequency of abdominal pain and stool abnormality without any effect on HP eradication rates. These findings provide further data regarding decreasing side effects of HP treatment regimens with the addition of clidinium-C. This issue needs further studies.

## Notes

**Funding: **The study was supported by Golestan University of Medical Sciences.
